# Pulmonary fibrosis: pathogenesis and therapeutic strategies

**DOI:** 10.1002/mco2.744

**Published:** 2024-09-23

**Authors:** Jianhai Wang, Kuan Li, De Hao, Xue Li, Yu Zhu, Hongzhi Yu, Huaiyong Chen

**Affiliations:** ^1^ Department of Respiratory Medicine Haihe Hospital Tianjin University Tianjin China; ^2^ Department of Tuberculosis Haihe Hospital Tianjin University Tianjin China; ^3^ Key Research Laboratory for Infectious Disease Prevention for State Administration of Traditional Chinese Medicine Tianjin Institute of Respiratory Diseases Tianjin China; ^4^ Tianjin Key Laboratory of Lung Regenerative Medicine, Haihe Hospital Tianjin University Tianjin China; ^5^ Department of Clinical Laboratory Nankai University Affiliated Third Central Hospital Tianjin China; ^6^ Department of Clinical Laboratory The Third Central Hospital of Tianjin Tianjin Key Laboratory of Extracorporeal Life Support for Critical Diseases Artificial Cell Engineering Technology Research Center of Tianjin Tianjin Institute of Hepatobiliary Disease Tianjin China

**Keywords:** biomarkers, endothelium, epithelium, immune cells, microbiome

## Abstract

Pulmonary fibrosis (PF) is a chronic and progressive lung disease characterized by extensive alterations of cellular fate and function and excessive accumulation of extracellular matrix, leading to lung tissue scarring and impaired respiratory function. Although our understanding of its pathogenesis has increased, effective treatments remain scarce, and fibrotic progression is a major cause of mortality. Recent research has identified various etiological factors, including genetic predispositions, environmental exposures, and lifestyle factors, which contribute to the onset and progression of PF. Nonetheless, the precise mechanisms by which these factors interact to drive fibrosis are not yet fully elucidated. This review thoroughly examines the diverse etiological factors, cellular and molecular mechanisms, and key signaling pathways involved in PF, such as TGF‐β, WNT/β‐catenin, and PI3K/Akt/mTOR. It also discusses current therapeutic strategies, including antifibrotic agents like pirfenidone and nintedanib, and explores emerging treatments targeting fibrosis and cellular senescence. Emphasizing the need for omni‐target approaches to overcome the limitations of current therapies, this review integrates recent findings to enhance our understanding of PF and contribute to the development of more effective prevention and management strategies, ultimately improving patient outcomes.

## Graphical abstract

This figure illustrates the interconnected and mutually reinforcing relationship between the study of mechanisms, diagnosis, and treatment of PF. The cycle begins with understanding the mechanisms underlying PF, which leads to the formulation of novel hypotheses. These hypotheses drive the development of new diagnostic tools and classification efforts, enhancing the ability to accurately identify and treat the disease. Insights gained from diagnostics inform therapeutic strategies, leading to novel interventions that improve patient outcomes. As new therapies are implemented, they provide feedback that refines our understanding of mechanisms and diagnostics, thus perpetuating the cycle of innovation and improvement. Throughout this process, various omics technologies are utilized to explore and elucidate the complexities of PF, ensuring a comprehensive and detailed approach to its study and management

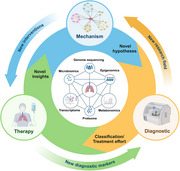

## 1 INTRODUCTION

Pulmonary fibrosis (PF) is a chronic, progressive lung disease marked by excessive accumulation of extracellular matrix (ECM) components, resulting in lung tissue scarring and impaired respiratory function.^1^ Idiopathic pulmonary fibrosis (IPF) is the most common form of PF, with its prevalence rising globally in recent years. The annual incidence of IPF is estimated to be 0.57–4.51 cases per 100,000 in Asia, 0.33–2.51 cases in Europe, and 2.4–2.98 cases in North America.^2^ IPF is more frequent in males and uncommon in individuals under 50, with a median diagnosis age of approximately 65 years.^3^ The disease course is unpredictable, with a poor prognosis and an average survival of 2−3 years. Acute exacerbations may occur, significantly increasing mortality.^4^

Despite extensive research, several gaps remain in the understanding of PF. The exact mechanisms by which genetic predisposition and environmental exposure synergize to initiate and propagate fibrotic processes have not yet been fully elucidated. The role of immune responses, particularly the contribution of different immune cell subsets, in modulating fibrosis requires further investigation. Current therapeutic strategies for PF primarily focus on the use of anti‐inflammatory and antifibrotic agents. Although these treatments can slow disease progression, they do not reverse established fibrosis and are often associated with significant side effects. Therefore, innovative therapies that target the underlying mechanisms of PF and offer better efficacy and safety profiles are urgently needed.

Recent studies have identified several key factors that contribute to the development of PF. Occupational and environmental exposures, including the inhalation of harmful substances such as asbestos, silica dust, and organic antigens, are significant contributors. These agents directly injure the lung tissue, triggering chronic inflammation and fibrotic processes.^5^ Additionally, lifestyle factors, such as smoking and chronic alcohol consumption, are strongly correlated with an increased risk of PF.^6^ Smoking introduces toxic constituents into the lungs, causing oxidative stress and inflammation that promote fibrotic changes. Chronic alcohol consumption impairs the natural repair mechanisms of the lungs, predisposing individuals to fibrosis.^6^ Preexisting medical conditions such as gastroesophageal reflux disease (GERD) and chronic viral infections also play critical roles in the pathogenesis of PF by perpetuating chronic inflammation and lung tissue damage.^7^, ^8^

The pathogenesis of PF involves a complex interplay of various cellular and molecular mechanisms. At the cellular level, interactions among epithelial cells, fibroblasts, immune cells, and endothelial cells (ECs) are crucial for driving the fibrotic process.^9^ Alveolar epithelial cells, particularly type II alveolar cells (AT2), play a significant role in lung repair and regeneration.^10^ However, repeated injury to these cells can lead to aberrant repair mechanisms, resulting in the differentiation of fibroblasts into myofibroblasts, which are central to the fibrotic response.^11^, ^12^ These myofibroblasts produce excessive ECM components, leading to scarring and stiffening of lung tissue. The molecular pathways implicated in PF include the TGF‐β/Smad, WNT/β‐catenin, and PI3K/Akt/mTOR signaling pathways. These pathways regulate various aspects of cell proliferation, differentiation, and apoptosis and contribute to the progression of fibrosis.

This review first examines the etiological and risk factors associated with PF, including genetic and environmental influences. It then explores the cellular and molecular mechanisms underlying the disease, with an emphasis on key signaling pathways. Subsequently, the review assesses current diagnostic tools, biomarkers, and therapeutic strategies, including emerging treatments. Finally, it discusses future research directions, highlighting the need for innovative, multitargeted approaches for effective disease management. By integrating the latest research and identifying critical gaps, this review aims to connect academic research with clinical practice, advancing the understanding and treatment of PF.

## 2 PATHOGENESIS OF PF

**FIGURE 1:**
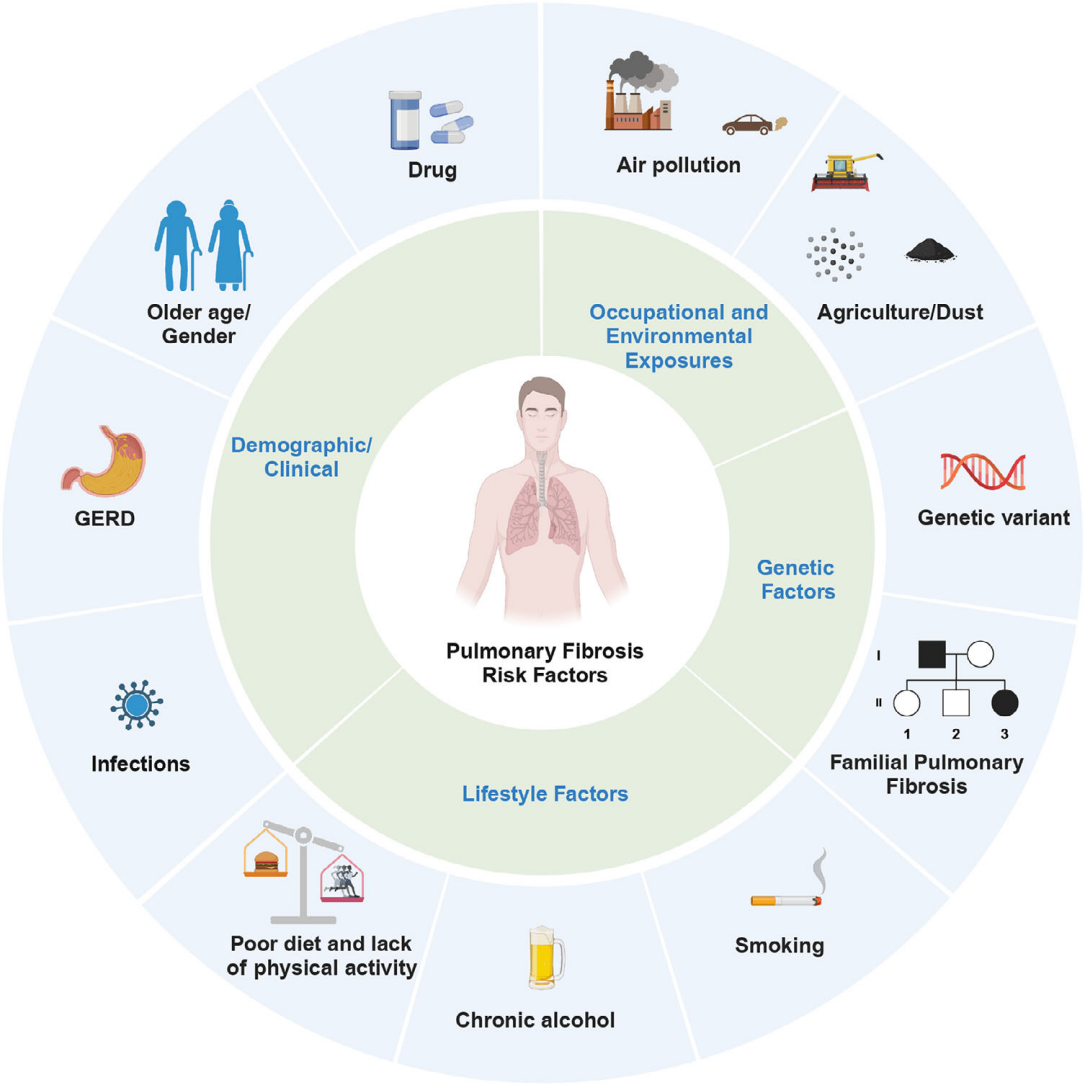
PF risk factors. This diagram illustrates the multifactorial risk factors associated with PF, categorized into four main groups: demographic/clinical, occupational and environmental exposures, genetic factors, and lifestyle factors. This figure was designed using BioRender (https://biorender.com/).

The etiology of PF involves a complex interplay between genetic factors, environmental exposure, and underlying medical conditions (Figure 1). A comprehensive understanding of these risk factors is crucial to develop targeted prevention and management strategies for this debilitating disease. This section discusses the multifaceted etiological factors, cellular and molecular mechanisms, inflammatory and fibrotic processes, and roles of various cell types involved in PF.

### 2.1 Etiology and risk factors

#### 2.1.1 Occupational and environmental exposures

Occupational and environmental exposures are significant contributors to PF. Inhalation of harmful substances such as asbestos, silica dust, and organic antigens can trigger chronic lung inflammation and fibrosis.^13^, ^14^ These agents directly injure the lung tissue, initiating a persistent inflammatory response. For example, workers in construction and mining are at higher risk because of their exposure to silica dust, which can lead to silicosis and subsequent PF.^15^ Similarly, exposure to asbestos, which is commonly found in older buildings, can cause asbestosis, a condition strongly associated with PF.^16^ Additionally, certain agricultural exposures, such as the inhalation of moldy hay (farmer's lungs) or bird droppings (bird fancier's lungs), can lead to hypersensitivity pneumonitis, which may progress to PF if not properly managed.^17^ Environmental pollutants such as traffic‐related air pollution have also been implicated in the development of PF.^18^, ^19^, ^20^ These pollutants induce oxidative stress and inflammation, contributing to lung tissue damage and fibrosis.

#### 2.1.2 Lifestyle factors

Lifestyle factors significantly influence the risk of PF. Smoking is a major risk factor for PF, and studies have shown a strong correlation between cigarette smoke exposure and PF development.^21^, ^22^ The toxic constituents of cigarette smoke, including reactive oxygen species (ROS) and proinflammatory agents, cause direct lung tissue damage and induce chronic inflammatory processes. Inflammation and tissue damage create a favorable environment for fibrosis.^23^ Chronic alcohol consumption has also been linked to PF.^24^ Alcohol impairs the natural repair mechanisms of the lungs, making them more susceptible to fibrosis. Alcohol‐induced oxidative stress and inflammation are thought to play important roles in this process.^25^ Additionally, lifestyle factors such as poor diet and lack of physical activity, which contribute to poor overall health, can exacerbate the risk of developing PF.

#### 2.1.3 Demographic factors and comorbidities

Demographic and clinical factors are associated with an increased risk of PF. Age is a significant risk factor, with PF most commonly diagnosed in individuals aged 50−70 years.^26^ Additionally, sex plays a role as IPF, the most common type, is more prevalent in men than in women.^27^, ^28^ GERD is a notable example. It can lead to the microaspiration of stomach contents into the lungs, causing chronic irritation and inflammation, which can result in fibrotic changes over time. Chronic inflammation due to microaspiration is thought to damage the lung epithelium and promote fibrosis.^29^ Moreover, chronic viral infections have been implicated in PF pathogenesis.^30^, ^31^ Hepatitis C and Epstein–Barr virus infections are notable examples. These viruses can persist in the body and cause chronic immune activation and inflammation, contributing to lung tissue damage and fibrosis.^32^, ^33^ Additionally, other chronic infections and autoimmune diseases, such as rheumatoid arthritis and systemic sclerosis, are associated with a higher incidence of PF, suggesting a link between chronic systemic inflammation and lung fibrosis.^34^

#### 2.1.4 Genetic susceptibility

Genetic susceptibility to idiopathic IPF has been increasingly elucidated through genome‐wide association studies (GWAS) and whole‐exome sequencing. The *MUC5B* promoter polymorphism (rs35705950) is highly prevalent among patients with IPF and is significantly associated with disease development by altering mucin production, potentially leading to aberrant epithelial repair and fibrosis.^35^, ^36^ Telomere‐related genes such as *TERT*, *TERC*, and *RTEL1* are also critical, with variants linked to both familial and sporadic forms of PF through telomere shortening and chromosomal instability.^37^, ^38^, ^39^ Mutations in surfactant protein genes, such as *SFTPC* and *SFTPA2*, disrupt surfactant metabolism, leading to increased lung injury and fibrosis.^40^, ^41^, ^42^, ^43^ GWAS have identified additional susceptibility loci, including *TOLLIP* and *DSP*, that affect the immune response and cell adhesion pathways.^44^, ^45^, ^46^ Other implicated genes include *HLA*, *AKAP13*, *SPPL2C*, and *KIF15*, highlighting their roles in immune regulation, profibrotic signaling, and mitotic spindle assembly in IPF.^47^, ^48^, ^49^, ^50^

Familial aggregation significantly affects the progression and prognosis of IPF, with familial cases often presenting earlier and more aggressively than sporadic cases.^51^ Genetic predisposition, particularly involving telomere‐related genes, complicates treatment strategies because of varying responses to conventional therapies.^52^, ^53^, ^54^ Genetic screening and counseling are essential for early diagnosis and personalized treatment to improve disease management and outcomes.

### 2.2 Fibrotic progression

The etiology of PF remains elusive; however, significant advancements have been made in understanding the underlying mechanisms of this chronic lung disease. Current knowledge indicates that PF arises from progressive dysfunction due to abnormal repair mechanisms triggered by repetitive injury to alveolar epithelial cells.^12^, ^55^ These microinjuries primarily affect AT2 cells and provoke immune or inflammatory responses in lung immune cells, exacerbating damage to both epithelial and ECs.^56^ This cascade of events leads to the reprogramming of affected epithelial cells, causing them to secrete numerous profibrotic factors and become abnormally activated. This process compromises the epithelial integrity and activates aberrant repair mechanisms and stress response pathways, culminating in the differentiation of fibroblasts into myofibroblasts.^11^

The pathogenesis of PF can be conceptualized into three main stages: immune and inflammatory responses in the alveoli, alveolar repair, and fibrosis. The cumulative effects of these stages, including inflammation, tissue damage, and repair, ultimately lead to fibrotic changes.^57^ Environmental factors such as dust and other harmful substances contribute to this process by causing injury to alveolar epithelial cells and capillary ECs. This injury disrupts the basement membrane, leading to the abnormal proliferation of AT2 and ECs. This results in the failure of re‐epithelialization and re‐endothelialization of the alveolar–capillary barrier, collapse of the alveoli, formation of fibrous clots, proliferation of fibroblasts, and transformation of fibroblasts into myofibroblasts.^58^

Moreover, damaged lung epithelium loses its ability to regulate fibroblast proliferation and matrix deposition, leading to excessive ECM accumulation and PF development.^59^ The intricate interplay between these cellular responses underscores the complexity of PF pathogenesis, in which immune responses, environmental factors, and abnormal cellular repair mechanisms converge to drive fibrotic processes.

### 2.3 Cellular contributions to PF

**FIGURE 2:**
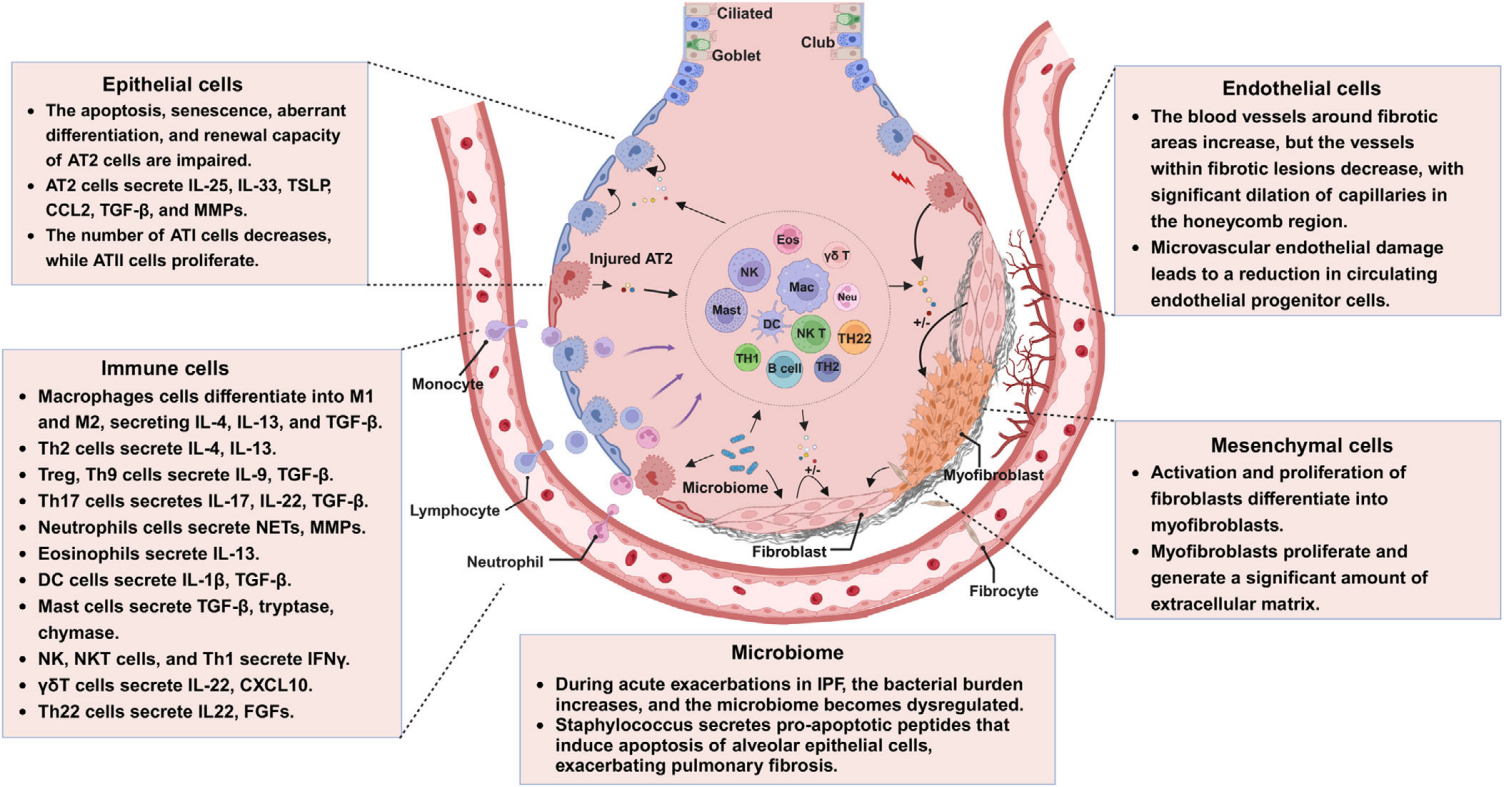
Cellular and microbiome contributions to PF. This schematic illustrates the various cell types and microbiome components involved in the pathogenesis of PF, highlighting their roles and interactions within the lung microenvironment. This figure was designed using BioRender (https://biorender.com/).

The pathogenesis of PF involves a complex interplay between various cell types and the microbiome. This section highlights the roles of mesenchymal, epithelial, immune, and ECs and the microbiome in PF (Figure 2).

#### 2.3.1 Lung fibroblasts

In PF, lung fibroblasts produce significant amounts of collagen, crucial for disease progression. Key cell types include fibroblasts, myofibroblasts, and lipofibroblasts. Advances in single‐cell sequencing have revealed that multiple mesenchymal cell subtypes have distinct gene expression profiles and functions.

Fibroblasts, which are derived from embryonic mesenchymal cells, are distributed throughout the body and are essential for tissue repair and local cellular response regulation. Fibroblast activation in the lungs involves cell proliferation, migration, and ECM production.^60^ This activation is significant in PF, in which ECM deposition is a fundamental pathological feature. Since the mid‐1990s, several studies have highlighted the role of alveolar fibroblasts in alveolar epithelial proliferation and differentiation.^61^ Alveolar fibroblasts provide and modify ECM scaffolds, generate tensile forces for septal wall extension, and deliver paracrine signals essential for cellular proliferation and differentiation.^61^ During PF, fibroblasts are activated by cytokines, growth factors, and mechanical stress, leading to their differentiation into myofibroblasts. The continuous activation and proliferation of myofibroblasts contribute to PF progression.^62^ Notably, inhibition of fibroblast differentiation can improve fibrosis. For instance, TGF‐β induces high MBD2 expression, promoting fibroblast differentiation into myofibroblasts and leading to PF.^63^ Fibroblasts from patients with IPF are notably more invasive and potentially driven by HER2 signaling, which enhances fibroblast invasiveness and worsens PF.^64^

Myofibroblasts were first identified in rat wound granulation tissue and exhibited characteristics of both fibroblasts and smooth muscle cells.^65^ They play an essential role in rapid tissue repair by depositing collagen scars.^62^ In PF, myofibroblasts originate from resident fibroblasts, epithelial‐to‐mesenchymal transition (EndMT), and circulating fibrocytes, leading to excessive fibrosis and impaired lung function.^66^, ^67^ The inhibition of myofibroblast function can reduce fibrosis.^68^, ^69^

Lipofibroblasts, which were first identified in rat lungs, contain substantial lipid bodies and are located near AT2 cells.^70^ Their presence in human lungs remains controversial. Lipofibroblasts store and synthesize vitamin A, contribute to surfactant production, and play a vital role in alveolar development. Recent research suggests that transforming lipofibroblasts into myofibroblasts contributes to the pathogenesis of experimental lung fibrosis and the reversal of ECM deposition, highlighting fibroblast phenotype modification as a therapeutic approach.^71^ During lung fibrosis, lipofibroblasts transform into myofibroblasts through TGF‐β1 signaling. These activated myofibroblasts accumulate in fibrotic regions, producing large amounts of ECM proteins, particularly collagen, leading to the destruction of the lung architecture and impaired gas exchange. Some myofibroblasts can dedifferentiate back into a lipofibroblast‐like phenotype during fibrosis resolution, regulated by PPARγ signaling.^72^ Promoting adipogenic differentiation of myofibroblasts can combat persistent PF, as demonstrated by omentin‐1′s ability to reverse established PF.^73^

Advancements in single‐cell sequencing have revealed several new fibroblast subtypes, each of which has a distinct role in PF. Xie et al.^74^ identified three subtypes: Col13a1 matrix fibroblasts, Col14a1 matrix fibroblasts, and mesenchymal progenitors. The proportion of matrix fibroblasts in fibrotic mesenchymal cells increased from 30 to 50%. Mesenchymal progenitors differentiate into lipofibroblasts and Col14a1 matrix fibroblasts. Additionally, PDGFRB^high^ fibroblasts, which are significantly increased in fibrotic lungs, contribute to ECM production by upregulating ECM‐related genes and differentiating into Col13a1 and Col14a1 matrix fibroblasts. Liu et al.^75^ identified a novel mesenchymal subpopulation characterized by Ebf1, which has a unique transcriptomic signature. In the embryonic lungs, this population coexpresses pericyte markers. In adult and fibrotic lungs, Ebf1+ populations diverge into groups with pericyte markers or unique signatures. A similar mesenchymal population has been identified in the human postnatal lung, along with a distinct pericyte cluster. Lee et al.^75^ identified that p16INK4a^+^ fibroblasts play a role in the formation of pathological fibroblast subsets in IPF.

#### 2.3.2 Epithelial cells

There are various types of lung epithelial cells, including AT2, alveolar type 1 (AT1), club, and basal cells, each playing distinct roles in lung function and contributing differently to fibrosis.

The alveolar epithelium, which is essential for gas exchange, comprises AT1 and AT2 cells. AT1 cells cover approximately 96% of the alveolar surface area, but are fewer in number owing to their large, flattened shape, which facilitates efficient gas exchange.^10^ In contrast, AT2 cells cover only 4% of the surface but constitute 60% of alveolar epithelial cells, producing pulmonary surfactants that reduce surface tension and prevent lung collapse.^76^, ^77^ AT2 cells also act as progenitor cells that are crucial for alveolar repair and regeneration.^10^, ^78^ Upon injury, AT2 cells differentiate into AT1 and new AT2 cells, aiding epithelial restoration.^79^, ^80^ AT2 cell dysfunction is the key driver in PF. Senescence rather than the loss of these cells leads to fibrosis, with p53 activation playing a significant role.^56^ Damaged AT1 cells prompt AT2 cells to differentiate, a process involving transitional cells such as prealveolar type 1 transitional state cells, which are prone to DNA damage and show enriched TP53 and TGFβ signaling.^81^ Damage‐associated transitional progenitors also differentiate into AT1 cells after lung injury, with similar cells found in IPF tissue samples.^82^

Club cells, which are nonciliated bronchiolar epithelial cells, are involved in xenobiotic metabolism, immune regulation via club cell secretory proteins, and progenitor cell activity, which is essential for epithelial repair.^82^, ^83^ Club cells play a significant role in IPF pathogenesis.^84^, ^85^ In IPF, club cells undergo phenoconversion, losing their typical morphology and acquiring the properties of alveolar epithelial cells, including the upregulation of proteins such as Claudin 10.^84^, ^85^ This phenomenon is associated with the initiation and progression of IPF, as phenoconverted club cells exhibit pleomorphism, migrate to alveolar regions, and contribute to alveolar bronchiolization and fibroblastic foci development.^84^, ^86^ Additionally, club cells activate fibroblasts paracrinally by secreting factors such as TGF‐β, playing a crucial role in fibrosis. Knockout models have demonstrated that ablation of key genes such as *PDCD5* in club cells significantly reduces fibrotic marker expression and fibrosis development.^87^

Basal cells, known as KRT5^+^ epithelial progenitors, are essential for maintaining and repairing the pseudostratified epithelium of the respiratory tract.^88^ They respond to epithelial injury by differentiating into various specialized cell types, such as ciliated and secretory cells, and by maintaining a reservoir of progenitor cells.^89^, ^90^ In IPF, basal cells undergo notable changes, including hyperplasia and honeycomb cyst formation, which are characteristic of advanced IPF.^91^ Single‐cell RNA sequencing (scRNA‐seq) has revealed distinct basal cell subtypes in healthy and fibrotic lungs, highlighting their heterogeneity and dynamic responses to fibrotic cues.^92^, ^93^, ^94^ In normal lungs, basal cells include multipotent progenitors and secretory‐primed basal cells (SPBs), the latter of which show similarities to basal cells in fibrotic tissues.^95^ This shift toward a secretory‐primed state is linked to mucociliary dysfunction and mucus overproduction in IPF.^96^ SPBs are abundant in regions expressing *MUC5B* within honeycomb cysts, connecting basal cell plasticity to the pathogenesis of fibrosis.^93^ Basal cells in IPF show increased expression of markers such as KRT14, VIM, and MMP7, indicating a transition to a mesenchymal‐like phenotype that contributes to fibrotic remodeling.^86^

#### 2.3.3 Immune cells

Immune response is a significant driver of fibrosis. The initial injury to AT2 cells triggers the release of various danger signals and cytokines, such as IL‐1β, TNF‐α, and IL‐6, which attract and activate immune cells. These immune cells, including macrophages, neutrophils, and lymphocytes, migrate to the site of injury and release additional proinflammatory mediators. This inflammatory milieu is essential for initiating tissue repair but can become chronic and pathological if unresolved.

Macrophages play a crucial role in the pathogenesis and progression of fibrotic lung disease through various mechanisms involving different subtypes and secretory profiles. Alveolar macrophages (AMs), interstitial macrophages (IMs), and monocyte‐derived macrophages are the primary populations involved in fibrotic processes.^97^, ^98^ AMs, typically maintaining lung homeostasis, become profibrotic under pathological conditions by producing TGF‐β, PDGF, FGF, and VEGF, which stimulate fibroblast proliferation and collagen synthesis. IMs contribute to fibrogenesis through interactions with structural cells and the secretion of profibrotic mediators such as CCL18.^99^ Subsets of IMs, such as Lyve1^hi^MHCII^lo^ IMs, aid in leukocyte chemotaxis and wound healing, whereas Lyve1^lo^MHCII^hi^ IMs are involved in antigen presentation and T cell activation.^100^ Monocyte‐derived macrophages infiltrate the lungs post‐injury and differentiate into profibrotic macrophages with high expression of fibrogenic genes such as *PDGFA* and *CSF1*, whose inhibition suppresses fibrosis.^101^, ^102^ Single‐cell transcriptomics have identified distinct macrophage populations in fibrotic lungs, including those expressing high levels of SPP1 and CHI3L1.^103^ The plasticity of macrophages allows them to adopt different phenotypes; in fibrosis, the M2‐like phenotype predominates, characterized by IL‐10 and TGF‐β secretion, promoting tissue repair and ECM production.^104^ Macrophages also regulate ECM synthesis and degradation through MMPs and their inhibitors, with the roles varying according to the MMP type.^97^ Novel subsets, such as SatMs, specifically exacerbate fibrosis.^105^ Understanding these roles will offer potential therapeutic avenues for mitigating fibrosis and improving patient outcomes.

Neutrophils are abundant in the lungs and bronchoalveolar lavage fluid (BALF) of patients with IPF and correlate with disease severity and poor prognosis. Elevated levels of neutrophil‐associated chemokines and proteins, such as interleukin‐8 (IL‐8) and S100A9, underscore the significant role of neutrophil‐driven inflammation in IPF.^106^ Neutrophils contribute to lung fibrosis by releasing neutrophil extracellular traps (NETs), which exacerbate tissue damage and promote fibrotic pathways while trapping and killing pathogens. NETs induce fibroblast differentiation into myofibroblasts, which are essential for collagen deposition and tissue remodeling, mediated by proteins such as neutrophil elastase (NE) and myeloperoxidase, which are found at elevated levels in patients with IPF.^107^ In mouse models of PF, the depletion of neutrophils or inhibition of their recruitment significantly reduce fibrosis. Mice lacking NE or those treated with NE inhibitors are protected from bleomycin‐induced fibrosis, a common IPF model. Additionally, the inhibition of formyl peptide receptor 1, which is crucial for neutrophil chemotaxis, mitigates fibrosis by reducing neutrophil infiltration.^108^ Neutrophils also secrete MMPs, which degrade ECM components and facilitate tissue remodeling, contributing to the aberrant repair and perpetuation of fibrosis in IPF.^109^ Moreover, neutrophils interact with other immune cells and fibroblasts to enhance fibrotic responses. Neutrophil‐derived leukotrienes promote the recruitment of additional inflammatory cells, amplifying the inflammatory and fibrotic cascade and underscoring the complexity of their role in IPF pathogenesis.

Lymphocytes play a multifaceted role in the pathogenesis of PF by engaging in both protective and pathological processes in the pulmonary microenvironment. In PF, the immune microenvironment is significantly altered, with elevated frequencies of memory B cells, plasma cells, and activated memory CD4^+^ T cells compared with those in healthy lungs, suggesting an enhanced adaptive immune response that may contribute to disease progression.^110^ T helper 2 cytokines, such as IL‐4, IL‐5, IL‐9, and IL‐13, produced by CD4^+^ T cells promote fibrosis by enhancing fibroblast activity and collagen deposition. Conversely, T regulatory cells (Tregs) and Th1 cells may exert antifibrotic effects through cytokines such as interferon‐gamma (IFN‐γ) and IL‐10.^111^, ^112^ B cells also contribute to fibrosis, with lymphoid aggregates composed of T, B, and dendritic cells present in IPF lungs, indicating a role for adaptive immune responses in fibrosis. B cells can differentiate into plasma cells that produce autoantibodies, potentially exacerbating tissue damage and fibrosis.^113^ Plasma cells and their antibodies are increasingly found in fibrotic lungs, indicating an ongoing immune response to self‐antigens or persistent infections.^114^ CD8^+^ T cells in IPF lungs show signs of activation and proliferation driven by chronic antigen exposure and inflammatory signals, contributing to tissue damage through cytotoxic activity and profibrotic cytokines such as TGF‐β.^115^, ^116^ The involvement of Tregs in IPF is complex, as they may either mitigate or exacerbate fibrosis depending on the context of their activation and cytokine production.^117^ The interplay between lymphocyte subsets and secreted factors underscores the complexity of immune regulation in patients with IPF. Targeting specific lymphocyte functions and associated signaling pathways may offer new therapeutic avenues for managing IPF, highlighting the need for a nuanced understanding of the role of immune cells in this chronic lung disease.

Group 2 innate lymphoid cells (ILC2s) contribute significantly to the pathogenesis of IPF. Activated by cytokines such as IL‐33, IL‐25, and TSLP, ILC2s produce type 2 cytokines, including IL‐5 and IL‐13, which are critical for the development of fibrosis.^118^ Studies in Regnase‐1‐deficient mice have shown that the absence of this post‐transcriptional regulator leads to ILC2 proliferation and activation, resulting in enhanced fibrosis. This is because Regnase‐1 normally degrades mRNAs encoding fibrosis‐related cytokines and transcription factors such as Gata3 and Egr1.^119^ In IPF, reduced IFN‐γ signaling leads to spontaneous ILC2 activation, promoting fibrosis. In Ifngr1^−/−^Rag2^−/−^ mice, the progression of fibrosis depends on IL‐33, with activated ILC2s inducing collagen production in fibroblasts, highlighting their direct role in fibrosis.^120^ Human studies have also shown that increased ILC2s in the peripheral blood and BALF correlate with disease severity and poor prognosis in patients with IPF, indicating a role similar to that in mouse models. Further research has shown that ILC2s, when activated by IL‐33, upregulate fibrosis‐associated genes and directly stimulate collagen production from fibroblasts. Coculture systems demonstrated that activated ILC2s significantly enhanced collagen deposition in fibroblasts.^119^ Depletion of ILC2s in mouse models reduces fibrosis, underscoring their essential role in the fibrotic process.^120^ In summary, ILC2s play a pivotal role in PF development and progression by producing profibrotic cytokines, interacting with fibroblasts, and contributing to collagen deposition. Regulating ILC2 activity, particularly through factors like Regnase‐1 and IFN‐γ signaling, represents a potential therapeutic target for mitigating fibrosis in patients with IPF.

#### 2.3.4 Endothelial cells

ECs play a critical role in PF pathogenesis. ECs contribute to fibrosis through EndMT, dysregulated angiocrine signaling, and interactions between fibroblasts and epithelial cells.^121^, ^122^ During EndMT, ECs acquire mesenchymal traits, thereby increasing the myofibroblast pool involved in ECM deposition. Yanagihara et al.^122^, ^123^ demonstrated that deleting matrix Gla protein in ECs activates TGF‐β signaling, promoting myofibroblast transition and worsening fibrosis. Conversely, inducing FoxA2 can reverse myofibroblasts back to ECs, suggesting its therapeutic potential in targeting EndMT.^122^, ^123^ EC–epithelial crosstalk is vital for lung homeostasis and repair. Chen et al.^124^ showed that lung ECs secrete angiocrine factors influencing AT2‐to‐AT1 cell differentiation, which is essential for lung repair, with Flt1 (VEGFR1) as the key regulator. Flt1 knockout in ECs reproduces the antifibrotic effects of microRNA‐200c and promotes epithelial trans‐differentiation, highlighting the importance of endothelial–epithelial signaling in mitigating fibrosis.^125^ Endothelial dysfunction and altered signaling pathways contribute to pulmonary vascular remodeling in PF. Loss of BMPR‐II signaling in ECs exacerbates endothelial dysfunction and promotes fibrogenesis through enhanced interactions with fibroblasts.^126^ scRNA‐seq has revealed the heterogeneity of EC populations, underscoring their complex roles in PF. These findings underscore the multifaceted roles of ECs in PF, suggesting that targeted therapies addressing endothelial dysfunction and signaling pathways could offer promising avenues for mitigating the progression of this debilitating disease.

### 2.4 Microbiome contribution

#### 2.4.1 Lung microbiome

The lung microbiome, which comprises diverse bacterial, viral, and fungal communities, significantly influences respiratory health and disease progression. Alterations in the lung microbiome have been observed in various lung diseases and have been shown to affect disease outcomes. The composition, diversity, and bacterial burden of the lung microbiome are crucial for maintaining homeostasis and influencing the disease state. In healthy individuals, the lung microbiome maintains a stable and diverse community structure by balancing bacterial migration, emigration, and replication. However, in lung diseases, such as PF, this balance is disrupted, leading to changes in microbial diversity and composition.^127^ Reduced microbial diversity (alpha diversity) and increased bacterial burden are associated with poor clinical outcomes and disease progression.^128^ Studies have found lower alpha diversity in patients with fibrotic lung conditions than in healthy controls, which is correlated with increased inflammation and lung fibrosis.^129^ Higher bacterial loads in the lower airways of patients with PF are associated with worse outcomes including higher mortality. This increased burden may result from enhanced bacterial migration or reduced bacterial clearance due to impaired mucociliary function. Specific bacterial taxa such as *Streptococcus* and *Staphylococcus* are associated with increased inflammation and tissue damage, exacerbating lung injury.^130^ The lung microbiome influences local immune responses, promoting either a protective or a pathogenic environment. Certain bacterial communities during fibrosis induce profibrotic immune responses, including Th17 cell activation and proinflammatory cytokine production.^127^ Disruptions in microbial communities can impair host defenses, increasing the susceptibility to infections and lung damage.^127^ These insights highlight the critical role of the lung microbiome in PF, suggesting that therapeutic strategies aimed at restoring the microbial balance could potentially mitigate disease progression and improve clinical outcomes.

#### 2.4.2 Gut microbiome

The gut microbiome influences various systemic diseases, including lung conditions, through the gut–lung axis, which is a bidirectional communication network linking the gastrointestinal and respiratory systems. Alterations in gut microbiota composition or dysbiosis are associated with the progression of several lung diseases by influencing immune responses and systemic inflammation. In mouse models of PF, distinct changes in the gut microbiome contribute to disease pathogenesis and progression. Gut dysbiosis exacerbates PF by increasing intestinal permeability, allowing microbial products, such as lipopolysaccharides (LPS), to enter the bloodstream, induce systemic inflammation, and augment fibrotic processes in the lungs.^131^ This systemic inflammation is mediated through Toll‐like receptor activation and the release of proinflammatory cytokines, perpetuating lung tissue remodeling and fibrosis.^131^ Gut microbiota‐derived metabolites modulate lung health. Short‐chain fatty acids (SCFAs) produced by fermenting dietary fibers possess anti‐inflammatory properties and may protect against fibrosis. Conversely, the depletion of beneficial SCFA‐producing bacteria and the accumulation of harmful metabolites enhance fibrotic responses. Notably, metabolites such as trigonelline, betaine, and cytosine are altered in fibrotic conditions, correlating with disease severity.^132^ Modulation of the gut microbiota through dietary interventions, probiotics, or antibiotics has shown promise in altering disease outcomes. Specific probiotics reduce lung inflammation and fibrosis in experimental models, suggesting that targeting gut dysbiosis may be a viable adjunctive strategy for managing PF.^132^ Mechanistic insights from 16S rDNA sequencing and metabolomics have revealed significant alterations in the gut bacterial taxa and metabolites in fibrotic models.^133^ Genera such as *Alloprevotella*, *Helicobacter*, *Rikenella*, and *Dubosiella* are correlated with fibrotic indicators, suggesting potential biomarkers for disease progression.^134^ Further research is necessary to translate these findings into clinical practice and to explore gut microbiota‐targeted therapies for managing PF.

### 2.5 Molecular pathways

#### 2.5.1 TGF‐β/Smad pathway

TGF‐β is a multifunctional cytokine regulating cellular processes including proliferation, differentiation, and apoptosis.^135^ It plays a critical role in PF, mainly by transforming fibroblasts into myofibroblasts, which are key for ECM production.^136^ The TGF‐β signaling pathway involves the phosphorylation of Smad proteins, which migrate to the nucleus to regulate genes linked to PF.^136^ TGF‐β also boosts the production of connective tissue growth factor (CTGF), augmenting fibrosis via increased ECM synthesis. It also inhibits ECM degradation by regulating MMPs and tissue inhibitors of metalloproteinases, leading to ECM accumulation.^135^, ^137^

Experimental models highlight TGF‐β’s significance in fibrosis progression, with its overexpression in mouse lungs causing substantial fibrosis.^138^ Conversely, inhibiting TGF‐β signaling mitigates fibrosis, suggesting therapeutic potential.^138^ Antifibrotic treatments targeting TGF‐β signaling pathways, including neutralizing antibodies and small molecule inhibitors, have demonstrated efficacy in preclinical trials.^139^ Furthermore, noncanonical TGF‐β signaling pathways involving mitogen‐activated protein kinases (MAPKs) and the phosphoinositide 3‐kinase (PI3K)/Akt pathway, along with interactions with other profibrotic mediators such as IL‐6 and angiotensin II, intensify fibrotic responses,^140^ underscoring the complexity of TGF‐β’s role in PF. Clinical evidence supports TGF‐β’s pivotal involvement in PF pathogenesis, as elevated levels have been detected in the BALF and lung tissues of patients with IPF.^141^ Genetic links have also been found, with polymorphisms in the *TGF‐β1* gene associated with increased susceptibility to IPF.^142^ Ongoing clinical trials focus on the efficacy of various TGF‐β inhibitors to reduce fibrosis and improve lung function, offering potential new therapeutic avenues that could change the course of this debilitating disease.^135^

#### 2.5.2 WNT/β‐catenin signaling

WNT signaling pathways are classified into canonical (β‐catenin‐dependent) and noncanonical (β‐catenin‐independent) pathways. In the context of PF, both pathways are implicated, although the canonical WNT/β‐catenin pathway has been more extensively studied. Activation of the canonical WNT pathway typically involves the binding of WNT ligands to Frizzled receptors and coreceptors, such as LRP5/6, leading to the stabilization and nuclear translocation of β‐catenin.^143^ This, in turn, results in the transcription of WNT target genes that promote fibrotic responses.^143^

Pirfenidone, an antifibrotic drug, alleviates PF by regulating the WNT/β‐catenin and TGF‐β/Smad pathways. Pirfenidone inhibits the activation of the WNT/β‐catenin pathway, which is otherwise upregulated in fibrotic lung tissues.^144^ By suppressing β‐catenin signaling, pirfenidone reduces the expression of fibrotic markers such as collagen and α‐SMA, thereby mitigating ECM deposition and fibrosis progression.^144^ Noncanonical WNT signaling pathways such as WNT5A and WNT11 promote myofibroblast differentiation and exacerbate fibrotic responses. This suggests that different branches of the WNT signaling network may contribute distinctively to the pathology of PF.^145^ Moreover, basal cell‐derived WNT7A plays a pivotal role in promoting fibrogenesis in the fibrotic niche of IPF.^146^ WNT7A signaling in the fibrotic microenvironment enhances fibroblast recruitment and activation, leading to increased collagen production and tissue stiffening.^146^ This finding underscores the importance of localized WNT signaling in modulating the fibrotic niche, and suggests that targeting specific WNT ligands may offer therapeutic benefits.

#### 2.5.3 PDGF signaling

PDGF is a homo‐ or heterodimeric molecule consisting of four different polypeptide chains (PDGF‐A, ‐B, ‐C, and ‐D) that interact with two types of PDGF receptors (PDGFR‐α and PDGFR‐β). These interactions result in receptor dimerization and autophosphorylation, activating several downstream signaling pathways, including Ras–MAPK, PI3K, and PLC‐γ, which regulate cell proliferation, migration, and survival.^147^ PDGF signaling has been shown to be upregulated in PF mouse models, suggesting its critical role in the disease pathology.^148^ Several studies have demonstrated the increased expression of PDGF and its receptors in fibrotic lung tissues. For instance, Zhao et al.^149^ reported elevated levels of PDGF‐A in a bleomycin‐induced PF mouse model. Additionally, Walsh et al.^150^ found increased PDGF‐BB and PDGF‐AA peptide levels in the BALF of rats treated with bleomycin, highlighting the role of these growth factors in promoting lung fibroblast growth. Similarly, both PDGF‐A and PDGF‐B at the mRNA and protein levels are increased in bleomycin‐treated mouse lungs.^151^

#### 2.5.4 PI3K/Akt and mTOR pathways

The PI3K/Akt pathway, upon activation by various stimuli, initiates a cascade of signaling events that promotes cell survival, proliferation, and metabolism. AKT, a central node in this pathway, phosphorylates and activates numerous downstream targets, including mTOR. The mTOR pathway, particularly mTOR complex 1 (mTORC1), is a crucial regulator of cellular growth and metabolism, influencing protein synthesis, autophagy, and lipid metabolism.^152^ Notably, the PI3K/Akt/mTOR pathway is significantly upregulated in PF.^153^ For example, LPS induces fibroblast proliferation and collagen synthesis through this pathway. LPS promotes aerobic glycolysis in lung fibroblasts via the PI3K/Akt/mTOR pathway, leading to increased collagen production. This metabolic shift, often referred to as the Warburg effect, supports the energetic and biosynthetic demands of proliferating fibroblasts and myofibroblasts in fibrotic tissues.^153^ Inhibitors targeting the PI3K/Akt/mTOR pathway have shown promise in preclinical PF models. For example, niclosamide ethanolamine salt alleviates PF by modulating the PI3K/mTORC1 pathway and reducing fibroblast proliferation and ECM deposition.^154^ Similarly, pharmacological inhibition of mTOR using agents such as rapamycin attenuates fibroblast activation and collagen synthesis, highlighting the therapeutic potential of targeting this pathway.^153^

#### 2.5.5 Hippo/Yes‐associated protein pathway

The Hippo/Yes‐associated protein (YAP) pathway involves a cascade of signaling proteins that regulate cell proliferation, apoptosis, and differentiation. When dysregulated, the Hippo pathway leads to the activation of the YAP and transcriptional coactivator with PDZ‐binding motif (TAZ), which translocates to the nucleus and drives the expression of profibrotic genes.^155^

In PF, YAP/TAZ activation is frequently observed in epithelial and mesenchymal cells. Notably, YAP/TAZ is significantly upregulated in fibrotic lung tissues.^156^ The crosstalk between YAP/TAZ and other signaling pathways, such as TGF‐β, is also vital. TGF‐β signaling is a major driver of fibrosis and can activate YAP/TAZ independently of the canonical Hippo pathway. This interaction creates a feedback loop that enhances the fibrotic response. For instance, YAP/TAZ can enhance the transcription of TGF‐β target genes, further promoting fibroblast activation and ECM production.^157^ Pharmacological inhibition of the Hippo/YAP pathway has shown promise in preclinical studies. Compounds which inhibit YAP/TAZ activity, such as verteporfin, have been shown to reduce fibrosis by preventing the nuclear translocation of these transcriptional coactivators.^158^ In addition, icariin, a natural flavonoid, attenuates bleomycin‐induced PF by targeting the Hippo/YAP pathway, thereby reducing fibroblast activation and ECM deposition.^159^ Moreover, the metabolic regulation of the Hippo/YAP pathway plays a role in fibrosis. Notably, glycolysis and lactate production influence YAP/TAZ activity. For instance, increased lactate levels in fibrotic lungs contribute to YAP/TAZ activation, which, in turn, drives the expression of profibrotic genes. This metabolic–epigenetic interaction highlights the complexity of fibrosis regulation and the potential for targeting metabolic pathways in conjunction with Hippo/YAP signaling.^160^, ^161^

#### 2.5.6 Notch signaling pathway

Notch signaling involves Notch receptors (Notch1−4) and their ligands (Jagged1, Jagged2, Delta‐like1, Delta‐like4), which facilitate cell‐to‐cell communication and transcriptional regulation upon activation.^162^ In PF, Notch signaling is aberrantly activated, contributing to the transition of various cell types such as fibroblasts and pericytes into myofibroblasts, which are pivotal for ECM deposition and tissue remodeling. Notably, Notch1 promotes pericyte‐to‐myofibroblast transition through the PDGFR/ROCK1 signaling pathway, enhancing fibroblast proliferation and differentiation, and exacerbating fibrosis.^163^

The crosstalk between Notch and TGF‐β signaling pathways amplifies the fibrotic response. TGF‐β upregulates Notch receptors and ligands, thereby enhancing Notch signaling activity. Additionally, Notch3 deficiency attenuates PF and impedes lung function decline, indicating the distinct roles of different Notch receptors in fibrosis.^164^ This highlights the importance of specific targeting of the Notch signaling pathway to achieve effective therapeutic outcomes.

#### 2.5.7 Oxidative stress

Oxidative stress plays significant roles in the pathogenesis of PF. ROS, primarily generated in the mitochondria and by NADPH oxidases (NOXs), are essential for normal cellular functions but can lead to tissue damage when produced in excess.^165^ In PF, excessive ROS production contributes to epithelial cell injury, fibroblast activation, and ECM remodeling, thereby exacerbating the fibrotic process.^165^ For instance, NOX2 and NOX4 are upregulated in PF, leading to increased ROS levels that activate pathways involving p53, Caspase‐3, and NF‐κB, promoting apoptosis and fibrosis.^165^ ROS‐induced damage in PF involves multiple mechanisms, including the induction of mitochondrial dysfunction, lipid peroxidation, DNA damage, and activation of profibrotic signaling pathways.^166^ ROS can enhance the activation of TGF‐β1 by promoting its release and activation from latent complexes in the ECM. This, in turn, stimulates myofibroblast differentiation and collagen production, further driving the fibrotic process.^165^ The role of oxidative stress is also evident in the upregulation of lung ornithine aminotransferase, which regulates mitochondrial ROS generation and TGF‐β1 activity, linking metabolic pathways to the fibrotic response.^167^ SLC15A3 plays a crucial role in regulating oxidative stress in macrophages. The deficiency of SLC15A3 protects against PF by enhancing the macrophage antioxidant stress response via the p62–NRF2 pathway.^168^ This regulation helps maintain homeostasis of the pulmonary microenvironment and inhibits the progression of fibrosis.

#### 2.5.8 Autophagy and apoptosis

Dysregulation of the autophagy and apoptosis pathways can lead to the accumulation of damaged cells and ECM components, contributing to fibrosis. Autophagy‐related proteins such as LC3 and Beclin‐1 have been shown to play roles in fibroblast activation and survival.^169^ Impaired autophagy can lead to accumulation of dysfunctional organelles and increased cellular stress, thereby promoting fibrosis. Selective deletion of Atg5 in AT2 cells resulted in defective alveolar epithelial repair and worsen fibrosis after bleomycin.^170^ Conversely, excessive apoptosis of epithelial cells can exacerbate tissue injury and inflammation, further promoting fibrosis.^171^

#### 2.5.9 Epigenetic modifications

Epigenetic modifications, including DNA methylation, histone modifications, and RNA‐based mechanisms, collectively regulate the transcriptional landscape of fibrotic lung tissues.

DNA methylation, particularly at CpG islands in gene promoters, plays a crucial role in silencing antifibrotic genes and promoting fibrotic gene expression. Aberrant DNA methylation patterns have been observed in IPF, including the hypermethylation of antifibrotic genes and the hypomethylation of profibrotic genes. This dysregulation promotes fibroblast activation and myofibroblast differentiation, key processes in ECM deposition and fibrosis progression.^172^

Histone modifications, including acetylation, methylation, and lactylation, also significantly contribute to PF. Histone lactylation, a relatively novel modification, induces a profibrotic phenotype in macrophages by upregulating genes such as *ARG1*, *OPN*, and *PDGFA*. This modification is mediated by p300, an acetyltransferase that facilitates lactylation of histone lysine residues and enhances the transcription of profibrotic genes.^173^ Increased lactate levels from augmented glycolysis in fibrotic lungs drive this process, highlighting the metabolic–epigenetic crosstalk in fibrosis.

RNA modifications, particularly N6‐methyladenosine (m6A) modifications, have been implicated in the development of PF.^174^ m6A modifications affect RNA stability and translation, influencing the expression of genes involved in fibrosis. For instance, the m6A reader protein YTHDC1 delays cellular senescence and PF by activating ATR, a critical DNA damage response protein, in an m6A‐independent manner. This suggests that RNA modifications play a role in the maintenance of cellular homeostasis and mitigation of fibrotic processes.^174^

## 3 CURRENT DIAGNOSTIC TOOLS AND BIOMARKERS

### 3.1 Radiological imaging

Radiological imaging is crucial in the diagnosis and assessment of PF. High‐resolution computed tomography (HRCT) is the gold standard for providing detailed images of the lung parenchyma and for identifying patterns characteristic of interstitial lung diseases (ILDs).^175^ HRCT is essential for diagnosing IPF, revealing specific patterns such as usual interstitial pneumonia.^9^ Although not routinely used for PF diagnosis, magnetic resonance imaging (MRI) offers functional imaging data, and advanced techniques, such as hyperpolarized gas MRI, enhance lung function assessment. Positron emission tomography (PET), particularly PET/computed tomography, assesses metabolic activity and inflammation to guide prognosis and therapy.^176^, ^177^ AI in radiological imaging is advancing, with algorithms analyzing HRCT scans to detect early fibrosis or disease progression, thereby improving the identification and quantification of fibrotic changes.^178^

### 3.2 Pulmonary function tests

Pulmonary function tests (PFTs) are essential for diagnosing and monitoring PF as they provide quantitative measures of lung function, which are crucial for assessing disease severity and progression. Specifically, forced vital capacity (FVC) is a key parameter in PFTs used to assess the restrictive lung defect characteristics of PF.^178^ A decline in FVC over time is a critical indicator of disease progression and is commonly used as a primary endpoint in clinical trials of PF treatment. Monitoring FVC helps clinicians evaluate the effectiveness of therapeutic interventions and make informed decisions regarding patient management.^178^ Additionally, the diffusing capacity of the lungs for carbon monoxide (DLCO) measures the ability of the lungs to transfer gas from inhaled air to the bloodstream. Reduced DLCO is a hallmark of PF and reflects an impaired gas exchange capacity due to fibrosis.^179^ Serial DLCO measurements provide valuable information regarding disease progression and treatment responses. The 6‐minute walk test (6MWT) assesses exercise tolerance and functional capacity in patients with PF by measuring the distance a patient can walk on a flat, hard surface in 6 min. This test is useful for evaluating the impact of PF on daily activities and overall functional status. A decline in the 6MWT distance is linked to worse outcomes and serves as a prognostic marker in clinical practice.^180^

### 3.3 Biomarkers

Biomarkers are pivotal in differentiating PF from other ILDs and assessing disease activity. These markers can be categorized based on their roles in various biological processes.

#### 3.3.1 Fibrogenesis and ECM remodeling markers

MMPs, particularly MMP‐1, MMP‐7, and MMP‐8, are significantly elevated in patients with PF and are involved in ECM degradation and remodeling.^181^, ^182^ MMP‐7, in particular, correlates with disease severity and progression, making it a promising biomarker.^183^ Elevated baseline MMP‐7 levels predict overall mortality and disease progression in untreated patients with IPF, independent of age, sex, smoking status, and lung function, as shown by a robust individual participant data meta‐analysis.^184^ Other markers such as periostin and osteopontin are also associated with fibrosis and ECM remodeling, reflecting ongoing fibrotic activity and matrix turnover.^185^, ^186^

#### 3.3.2 Alveolar epithelial cell injury markers

Levels of Krebs von den Lungen‐6 (KL‐6), surfactant proteins (SP‐A and SP‐D), and receptors for advanced glycation end‐products are elevated in patients with PF and reflect alveolar epithelial cell damage and repair processes.^141^, ^187^, ^188^ KL‐6 levels correlate with disease severity, extent of fibrosis, and prognosis. Elevated SP‐A and SP‐D levels are indicative of alveolar damage and inflammation in PF, and are used to differentiate PF from other lung diseases and assess disease progression.^189^

#### 3.3.3 Inflammatory and immune dysfunction markers

Markers such as YKL‐40, S100A8/A9, and CCL18 are elevated in PF and are involved in chronic inflammation and immune response dysregulation. These markers can indicate disease activity and predict disease exacerbation.^141^, ^190^ YKL‐40 is associated with macrophage activation and tissue remodeling, whereas S100A8, S100A9, and S100A12 serve as biomarkers for inflammation and fibrosis, offering potential targets for monitoring disease activity and prognosis.^141^ Elevated levels of these proteins in the peripheral blood correlate with disease progression.

Machine learning‐based approaches have identified differentially expressed genes such as *FHL2*, *HPCAL1*, *RNF182*, and *SLAIN1* as potential biomarkers for PF.^191^ These biomarkers were validated using various machine learning models, including LASSO logistic regression, support vector machine‐recursive feature elimination, and random forest algorithms. The integration of machine learning with biomarker discovery is a powerful tool for improving diagnostic accuracy and understanding disease mechanisms.^191^ scRNA‐seq and proteomic and metabolomic analyses have provided detailed insights into cellular heterogeneity by identifying specific cell populations and their associated gene expression profiles in PF lungs. These techniques help to identify cell‐specific biomarkers and understand the role of different cell types in disease progression.^192^

## 4 THERAPEUTIC STRATEGIES AND EMERGING TREATMENTS

### 4.1 Anti‐inflammation treatments

Traditional treatments for IPF include immunosuppressants and glucocorticoids. These therapies aim to reduce inflammation and immune system activity, which are hypothesized to mitigate fibrosis progression. However, extensive clinical studies have shown their limited efficacy and potential adverse effects, leading to a decline in their routine use.^5^ Immunosuppressants, such as azathioprine and cyclophosphamide, were initially used based on the belief that IPF has an autoimmune component. Similarly, glucocorticoids, such as prednisone, have been employed to suppress inflammation.^5^ However, current guidelines generally recommend against their use in IPF, except in specific situations, such as managing acute exacerbations.

### 4.2 Pirfenidone and nintedanib

Pirfenidone is an oral antifibrotic agent that inhibits the synthesis of TGF‐β and other cytokines, reducing fibroblast proliferation and collagen synthesis.^144^ Clinical trials have shown that pirfenidone reduces lung function decline and improves progression‐free survival (Table 1). It also possesses anti‐inflammatory and antioxidant properties that contribute to its therapeutic effects.^193^ Common side effects include gastrointestinal disturbances, photosensitive rashes, and elevated liver enzyme levels, which necessitate regular monitoring.^194^

**TABLE 1:** Summary of clinical trials on nintedanib from the past 5 years.

	Conditions	Phase	Treatment duration	Outcome
Kim et al.^195^	IPF	Phase 3	24 months	Overall survival and nonelective respiratory hospitalizations did not differ significantly between the pirfenidone and nintedanib groups.
Wijsenbeek et a.l^175^	PPF	Phase 3	24 months	Mitigated the progression of dyspnea, fatigue, cough, and ILD effects
Inoue et al.^196^	PF‐ILD	Phase 3	52 weeks	Lessen symptom progression and the impacts of PF‐ILD
Deterding et al.^197^	ChILD	Phase 3	24 weeks	In children and adolescents with fibrosing ILD, nintedanib dosing was safely tolerated.
Matteson et al.^198^	RA‐ILD	Phase 3	24 months	Slowed FVC decline, with few adverse events
Cottin et al.^199^	PF‐ILD	Phase 3	52 weeks	Immunomodulatory therapies did not affect the efficacy of nintedanib in reducing FVC decline.
Inoue et al.^200^	PF‐ILD	Phase 3	52 weeks	Slowed the progression of ILD in Japanese patients, slowed FVC decline
Schmid et al.^201^	PF‐ILD and SSc‐ILD	Phase 2/Phase 3	52 weeks	Nintedanib exposure correlated with ALT or AST elevations.
Kuwana et al.^202^	SSc‐ILD	Phase 3	52 weeks	Nintedanib slowed ILD progression in both Japanese and non‐Japanese patients with SSc‐ILD, with no subgroup heterogeneity.
Richeldi et al.^203^	IPF	Phase 3	24 weeks	Nintedanib slowed FVC decline and safety in IPF patients with different levels of gas exchange impairment.
Wells et al.^204^	PF‐ILD	Phase 3	52 weeks	Nintedanib reduces ILD progression, measured by FVC decline, in patients with chronic fibrosing ILD, regardless of diagnosis.
Song et al.^205^	IPF	Phase 3	52 weeks	Nintedanib sustainably slows IPF progression in Asian patients with acceptable long‐term safety and tolerability.
Moor et al.^206^	IPF	NA	24 weeks	Home monitoring did not improve overall HRQOL (K‐BILD) but tended to boost psychological well‐being and allowed tailored medication adjustments.

Abbreviations: PPF, progressive pulmonary fibrosis; PF‐ILD, progressive fibrosing interstitial lung disease; ChILD, childhood interstitial lung disease; RA‐ILD, rheumatoid arthritis develops interstitial lung disease; FVC, forced vital capacity; SSc‐ILD, systemic sclerosis‐associated ILD; NA, not applicable; HRQOL, health‐related quality of life; K‐BILD, king's brief interstitial lung disease questionnaire.

*Data resource*: https://www.clinicaltrials.gov.

Nintedanib is a tyrosine kinase inhibitor that targets multiple pathways involved in fibrosis, including VEGF, PDGF, and FGF receptors.^207^ It significantly slows the decline in FVC in patients with IPF by inhibiting fibroblast proliferation, migration, and transformation into myofibroblasts (Table 2).^207^ The side effects of nintedanib primarily include gastrointestinal symptoms, particularly diarrhea, and elevated liver enzyme levels, requiring dose adjustments and monitoring.^208^

**TABLE 2:** Summary of clinical trials on pirfenidone from the past 5 years.

	Conditions	Phase	Treatment duration	Outcome
Solomon et al.^209^	RA‐ILD	Phase 2	52 weeks	Slowed FVC decline, with few adverse events
Behr et al.^210^	PF‐ILD	Phase 2	48 weeks	Slow disease progression, a reduced decline in FVC
Behr et al.^211^	PF‐uILD	Phase 2	24 weeks	Pirfenidone is less effective in PF‐uILD patients on MMF but beneficial for those not on MMF, regardless of corticosteroid use. It was well tolerated with or without MMF and corticosteroids.
Sakamoto et al.^212^	IPF	Phase 3	48 weeks	Combining inhaled N‐acetylcysteine and pirfenidone may worsen IPF.
Behr et al.^213^	IPF	Phase 2	52 weeks	Adding sildenafil to pirfenidone offered no additional treatment benefits for advanced IPF patients with pulmonary hypertension risk, and no new safety issues were noted.
Blackwell et al.^214^	IPF	Phase 1	12 weeks	Pirfenidone is safe and well tolerated when combined with valganciclovir.
Blackwell et al.^215^	PF‐uILD	Phase 2	24 weeks	Pirfenidone may benefit patients with PF‐uILD and has an acceptable safety profile
Ikeda et al.^216^	IPF	Phase 3		Serum SP‐D was the key biomarker for pirfenidone efficacy in the IPF trial, with potential as a pharmacodynamic biomarker

Abbreviations: PF‐uILD, progressive fibrosing unclassifiable interstitial lung disease; MMF, mycophenolate mofetil.

*Data resource*: https://www.clinicaltrials.gov.

### 4.3 Supportive care

Pulmonary rehabilitation programs are designed to improve the physical and emotional wellbeing of patients with chronic respiratory diseases. These programs typically include exercise training, nutritional counseling, education on lung diseases, and breathing techniques.^217^ Pulmonary rehabilitation improves exercise capacity, reduces symptoms, and enhances the quality of life in patients with IPF.^217^ Oxygen therapy is prescribed to patients with IPF with resting or exertional hypoxemia to improve oxygenation and reduce the symptoms of breathlessness.^218^ Noninvasive ventilation may be used to support breathing in more advanced cases, particularly during acute exacerbations or in patients with concurrent sleep apnea.^219^ These interventions help to maintain adequate oxygen levels and reduce breathing.

### 4.4 Lung transplantation

Lung transplantation is considered for eligible patients with IPF and advanced disease refractory to medical therapy. This remains the only intervention that can significantly improve the survival and quality of life of selected patients. However, this procedure is associated with significant risks and requires lifelong immunosuppression to prevent organ rejection.^220^ Careful patient selection and management are essential to optimize patient outcomes.

### 4.5 Emerging treatments

#### 4.5.1 Targeting the immune response

Pharmacological interventions targeting the immune system in PF focus on modulating cytokines, immune checkpoints, and macrophages, and utilizing cellular therapies and precision medicine approaches. Cytokines, such as those targeting TGF‐β, IL‐6, IL‐1β, and IL‐13, play a central role in limiting fibrotic and inflammatory signaling pathway activities.^221^ For instance, fresolimumab inhibits TGF‐β, while tocilizumab and canakinumab target IL‐6 and IL‐1β, respectively. Monoclonal antibodies such as lebrikizumab and tralokinumab inhibit IL‐13 and reduce fibroblast activity and collagen deposition.^221^ Immune checkpoint inhibitors, including PD‐1/PD‐L1 inhibitors (nivolumab and pembrolizumab) and CTLA‐4 inhibitors (ipilimumab), have been explored for their potential to modulate immune responses and reduce fibrosis by enhancing the immune system activity against fibrotic tissues.^222^ Macrophage modulation involves the targeting of M2 macrophage polarization and monocyte recruitment.^223^ Agents such as dexamethasone modulate M2 macrophage activity, whereas cenicriviroc, a CCR2/CCR5 inhibitor, limits macrophage infiltration and reduces the fibrotic response.^105^ Cellular therapies, particularly T cell‐based therapies involving adoptive cell transfer or engineered T cells, aim to enhance the antifibrotic properties of immune cells.^224^ Precision medicine approaches utilize biomarker‐driven therapies and genetic/epigenetic modulation to tailor treatment to individual patient profiles. Advanced diagnostic techniques such as scRNA‐seq can identify specific cytokine profiles or immune cell populations for targeted interventions. Genetic and epigenetic therapies, including gene‐editing technologies and epigenetic drugs, aim to reprogram immune cells to reduce fibrosis by targeting the underlying factors that contribute to immune dysregulation. Overall, these multifaceted strategies aim to reduce inflammation, modulate immune responses, and slow or reverse the fibrotic process in patients with PF, thereby offering hope for improved patient outcomes. This comprehensive approach highlights the potential of immune‐targeted therapies to transform the treatment landscape for PF, addressing the complex interplay of immune mechanisms driving the disease, and paving the way for novel and effective treatments.

#### 4.5.2 Targeting fibrosis

Antifibrotic treatments for PF focus on key pathways that drive fibroblast activation, ECM accumulation, and collagen production.^225^ Transforming growth factor‐beta (TGF‐β) is a central mediator in fibrosis, and therapies targeting TGF‐β signaling, such as pamrevlumab, a monoclonal antibody against CTGF, reduce fibrosis and improve lung function.^226^ CTGF, induced by TGF‐β, is crucial in fibroblast activation and ECM production. The direct targeting of fibroblasts is another strategy. Integrin inhibitors, like those targeting integrin αvβ6, prevent the activation of latent TGF‐β, thereby reducing fibroblast activation and ECM deposition.^227^ Serine/threonine kinase inhibitors block the signaling pathways essential for fibroblast proliferation and myofibroblast differentiation, thereby hindering the fibrotic process.^228^, ^229^

ECM remodeling is another critical aspect of fibrosis. MMPs inhibitors regulate ECM turnover, prevent excessive matrix accumulation, and mitigate tissue remodeling, contributing to fibrosis progression.^230^ Targeting collagen production, a hallmark of fibrosis, can be achieved by inhibiting enzymes such as prolyl 4‐hydroxylase, thereby reducing collagen stability and accumulation.^231^

These antifibrotic strategies aim to disrupt the signals and processes that drive fibrosis in PF. By targeting TGF‐β signaling, fibroblast activation, ECM remodeling, and collagen production, these therapies show promise in slowing disease progression and improving patient outcomes. Continued research and clinical trials are essential to optimize these treatments and develop new agents to combat the fibrotic processes underlying PF, ultimately improving the quality of life of patients.

#### 4.5.3 Targeting senescence

Recent advances in the understanding of the pathophysiology of PF have highlighted the critical role of cellular senescence in driving fibrosis. Senescent cells exhibit a proinflammatory and profibrotic phenotype, known as the senescence‐associated secretory phenotype (SASP), which perpetuates tissue damage and fibrosis. Targeting cellular senescence has emerged as a promising therapeutic strategy for PF.^232^

Sirtuins, particularly SIRT3, play a significant role in modulating cellular senescence and fibrosis. Studies show that SIRT3 expression is markedly reduced in the lungs of patients with IPF and aged mice with persistent fibrosis.^233^ Restoring SIRT3 enhances fibrosis resolution by activating the FoxO3a transcription factor and re‐establishing susceptibility to apoptosis in myofibroblasts.^234^ SIRT3 activation mitigates epithelial cell senescence via the cGAS–STING pathway, thereby reducing fibrosis and improving lung function.^233^ Bone morphogenetic protein 4 (BMP4) inhibits PF by modulating senescence and mitophagy of lung fibroblasts. BMP4 administration in fibrotic mouse models decreases senescence markers and enhances mitophagy, leading to reduced fibrosis.^235^ Notably, senotherapeutics that target senescent cells to alleviate fibrosis show promise. For example, compounds such as navitoclax and dasatinib induce apoptosis in senescent cells and reduce fibrosis in mouse models.^232^ Inhibition of the SASP, which is characterized by proinflammatory cytokines and growth factors, also ameliorates fibrosis. Targeting key SASP components, such as IL‐1α, IL‐6, and TGF‐β, significantly reduces fibrosis and improves tissue regeneration.^232^ Enhancing mitophagy using pharmacological agents or genetic manipulation reduces senescence and fibrosis in lung fibroblasts. The activation of PINK1/Parkin‐mediated mitophagy decreases the expression of senescence and fibrotic markers.^235^ Additionally, honokiol, a natural compound, activates SIRT3 and reduces fibrosis via the cGAS‐STING pathway.^233^

#### 4.5.4 Targeting metabolism

PF is characterized by significant metabolic reprogramming, primarily driven by TGF‐β, which induces shifts toward increased glycolysis and mitochondrial dysfunction. This reprogramming process involves increased glycolytic enzyme expression, glucose transporter levels, and lactate production in fibroblasts, alveolar epithelial cells, and macrophages.^160^ Targeting these metabolic alterations may offer a novel therapeutic approach for PF.

Mitochondrial transplantation restores mitochondrial function, reverses the fibrotic phenotype, reduces glycolytic enzyme expression and lactate production, and increases ATP production.^236^ Modulation of lipid metabolism is another strategy, as dysregulated lipid metabolism contributes to PF. Promotion of fatty acid oxidation can mitigate fibrotic responses.^237^ Inhibiting glycolysis, using agents such as with 2‐deoxy‐d‐glucose (2‐DG) and targeting glycolytic regulators such as hypoxia‐inducible factor‐1 alpha, can reduce fibrosis.^238^, ^239^

Iron oxide nanoparticles enhance intercellular mitochondrial transfer, improve mitochondrial function, and reduce fibrosis.^240^ The modulation of macrophage metabolism can also reduce profibrotic activity.^241^ Addressing mitochondrial dynamics by promoting fusion and inhibiting excessive fission restores mitochondrial integrity and reduces fibrosis.^242^ These strategies offer promising avenues for the treatment of PF by targeting metabolic underpinnings.

#### 4.5.5 Cell‐based therapies

Cell‐based therapies for PF, focusing on the regeneration and repair of lung tissue through the transplantation of various cell types, have shown promising results. The most studied cell types used in these therapies include AT2, mesenchymal stromal cells (MSCs), induced pluripotent stem cells (iPSCs), and distal airway stem cells (DASCs). These therapies aim to restore lung function, reduce fibrosis, and improve the overall outcomes in patients with PF.

AT2 cells play a crucial role in maintaining alveolar homeostasis and repairing the damaged alveolar epithelium. Meta‐analyses have shown that AT2 transplantation significantly improves blood oxygen saturation, reduces lung hydroxyproline content, and decreases lung weight in mouse models of PF.^243^ These cells exhibit stem cell properties, allowing them to proliferate and transdifferentiate into AT1 cells, thereby restoring the integrity of the alveolar–capillary barrier and promoting lung regeneration.^243^

MSCs are another cell type that have been extensively investigated for their potential in the treatment of PF. MSCs can differentiate into multiple cell types, including alveolar epithelial cells and fibroblasts, and they secrete various bioactive molecules that modulate immune responses, reduce inflammation, and promote tissue repair. Notably, MSCs can attenuate AT2 cell senescence by regulating NAMPT‐mediated NAD metabolism, which is critical for maintaining cellular energy balance and reducing oxidative stress.^244^, ^245^ Furthermore, MSCs can inhibit myofibroblast differentiation of lung fibroblasts, thereby reducing fibrosis and improving lung function.^246^

iPSCs offer a versatile approach for PF treatment because of their ability to differentiate into any cell type. In mouse models of PF, iPSC‐derived alveolar epithelial cells have been shown to reduce fibrosis, restore lung structure, and improve lung function by modulating the WNT signaling pathway, which is essential for tissue repair and regeneration.^247^ Additionally, iPSCs can be used to generate patient‐specific cell lines, providing a personalized therapeutic approach and reducing the risk of immune rejection.

DASCs represent a relatively new focus in PF research. These cells can migrate to injured areas of the lung, where they can proliferate and differentiate into both alveolar and airway epithelial cells. DASCs have been shown to ameliorate bleomycin‐induced PF in mice by restoring the integrity of the lung epithelium and reducing inflammation and fibrosis.^248^ Their ability to self‐renew and differentiate into multiple lung cell types makes them promising candidates for regenerative therapy of PF.

Cell‐based therapies for PF offer a multifaceted treatment approach that targets different aspects of the disease process. AT2 cells focus on repairing and maintaining the alveolar epithelium; MSCs modulate the immune response and reduce fibrosis; iPSCs provide a versatile and personalized approach for lung regeneration; and DASCs offer robust regenerative potential. These therapies, either alone or in combination, hold significant promise for improving outcomes in patients with PF, moving closer to effective treatments and potentially reversing the progression of this debilitating disease. Further research and clinical trials are required to optimize these therapies, determine the best cell sources, and establish standardized protocols for their application in patients.

#### 4.5.6 Gene therapy

Gene therapy represents a promising avenue for treating PF by specifically targeting and modifying the key molecular pathways involved in disease progression. An innovative approach involves the use of adeno‐associated virus (AAV)‐mediated delivery systems. Notably, AAV9 vectors have been employed to deliver S1P lyase (SGPL1) directly into lung tissues, effectively reducing sphingosine‐1‐phosphate (S1P) levels. This reduction in S1P, a lipid mediator known to exacerbate fibrosis and inflammation, attenuates fibrotic signaling and decreases collagen deposition, thereby improving lung function in mouse models of BLM‐induced lung injury.^249^ Furthermore, AAV‐SGPL1 therapy reduces the expression of profibrotic markers such as TNFα, IL‐1β, fibronectin, and various collagen genes, demonstrating significant potential to modify the disease course by disrupting critical profibrotic pathways.^249^

Another promising strategy involves the combination of hepatocyte growth factor (HGF) with TGF‐β/Smad inhibitors, delivered via AAV9 vectors. This combination therapy has demonstrated efficacy in mouse models of silicosis by reducing fibrosis, promoting epithelial repair, and inhibiting myofibroblast differentiation. HGF facilitates the regeneration of lung epithelial cells, while TGF‐β/Smad inhibitors block pathways that contribute to fibrosis, resulting in synergistic effects that further ameliorate lung damage and improve respiratory function.^250^

Advancements in gene delivery technologies have also played crucial roles in enhancing the effectiveness of gene therapies for PF. Liposome‐based gene delivery systems have been developed to improve the uptake and expression of therapeutic genes in lung tissues. For instance, local administration of Plekhf1 gene therapy using liposomal carriers has shown the potential to reduce fibrotic responses and improve lung architecture by modulating the cellular mechanisms involved in fibrosis.^251^ In addition, perfluorocarbon nanoemulsions have been used to enhance the delivery of siRNAs targeting fibrotic genes. These nanoemulsions facilitate the efficient transport of siRNA into lung cells, thereby enhancing gene silencing and reducing fibrosis. The unique properties of perfluorocarbons, such as their ability to increase the bioavailability and stability of therapeutic siRNAs in the lung microenvironment, support their use in gene therapy.^252^

Overall, gene therapy offers a multifaceted approach to combat PF by addressing the underlying molecular mechanisms that drive fibrosis. AAV vectors, particularly AAV9, provide a versatile platform for delivering therapeutic genes with high specificity and sustained expression. The combination of gene therapies targeting multiple pathways, along with advanced delivery systems, such as liposomal carriers and nanoemulsions, holds significant promise for improving clinical outcomes in patients with PF. As research continues, these approaches are expected to be refined and optimized, paving the way for effective gene‐based treatments that can halt or even reverse PF progression. Further studies and clinical trials are necessary to validate the efficacy and safety of these therapies in patients, ultimately aiming to provide hope for those suffering from this debilitating condition.

#### 4.5.7 Traditional Chinese medicine

Traditional Chinese medicine (TCM) has been explored as a therapeutic approach for PF, and several studies have highlighted its potential benefits. One such formulation, Bufei Huoxue (BFHX) capsules, comprises components of Astragalus membranaceus, Paeonia lactiflora, and Psoralea corylifolia and has shown efficacy in alleviating PF through various mechanisms.^253^ In a mouse model of bleomycin‐induced PF, BFHX treatment significantly reduced fibrosis, improved lung function, and decreased inflammation. Treatment with BFHX led to a notable reduction in IL‐6 and TNF‐α levels, which are key inflammatory cytokines implicated in the pathogenesis of PF.^253^ Additionally, BFHX treatment upregulated the expression of E‐cadherin and downregulated α‐SMA, collagen I, vimentin, and fibronectin, indicating its role in inhibiting ECM deposition. The underlying mechanism involves the inhibition of the TGF‐β1/Smad2/3 signaling pathway, which is crucial for the transition of fibroblasts to myofibroblasts and the progression of fibrosis.^253^

Other TCM compounds have demonstrated potential antifibrotic effects. Similarly, Psoralen, a compound isolated from P. corylifolia, attenuates fibroblast activation and collagen deposition.^254^ These findings suggest that TCM formulations, such as BFHX and its individual components, offer a multifaceted approach to combating PF by modulating critical pathways involved in fibrosis and inflammation.

Studies have explored the combined use of TCM and conventional pulmonary drug delivery systems to enhance therapeutic outcomes. For example, integrating TCM formulations with inhalation therapies can improve drug bioavailability and target specific lung regions affected by fibrosis. This approach not only leverages the therapeutic benefits of TCM but also enhances the precision and efficacy of treatment modalities for PF.^255^

In summary, TCM formulations such as BFHX capsules hold significant promise for PF treatment. By targeting key inflammatory and fibrotic pathways, these formulations reduce fibrosis, improve lung function, and enhance overall patient outcomes. Future research should focus on elucidating the molecular mechanisms of TCM, optimizing delivery systems, and conducting large‐scale clinical trials to fully elucidate the therapeutic potential of these ancient remedies in modern medicine.

## 5 CONCLUSION AND PROSPECTS

**FIGURE 3:**
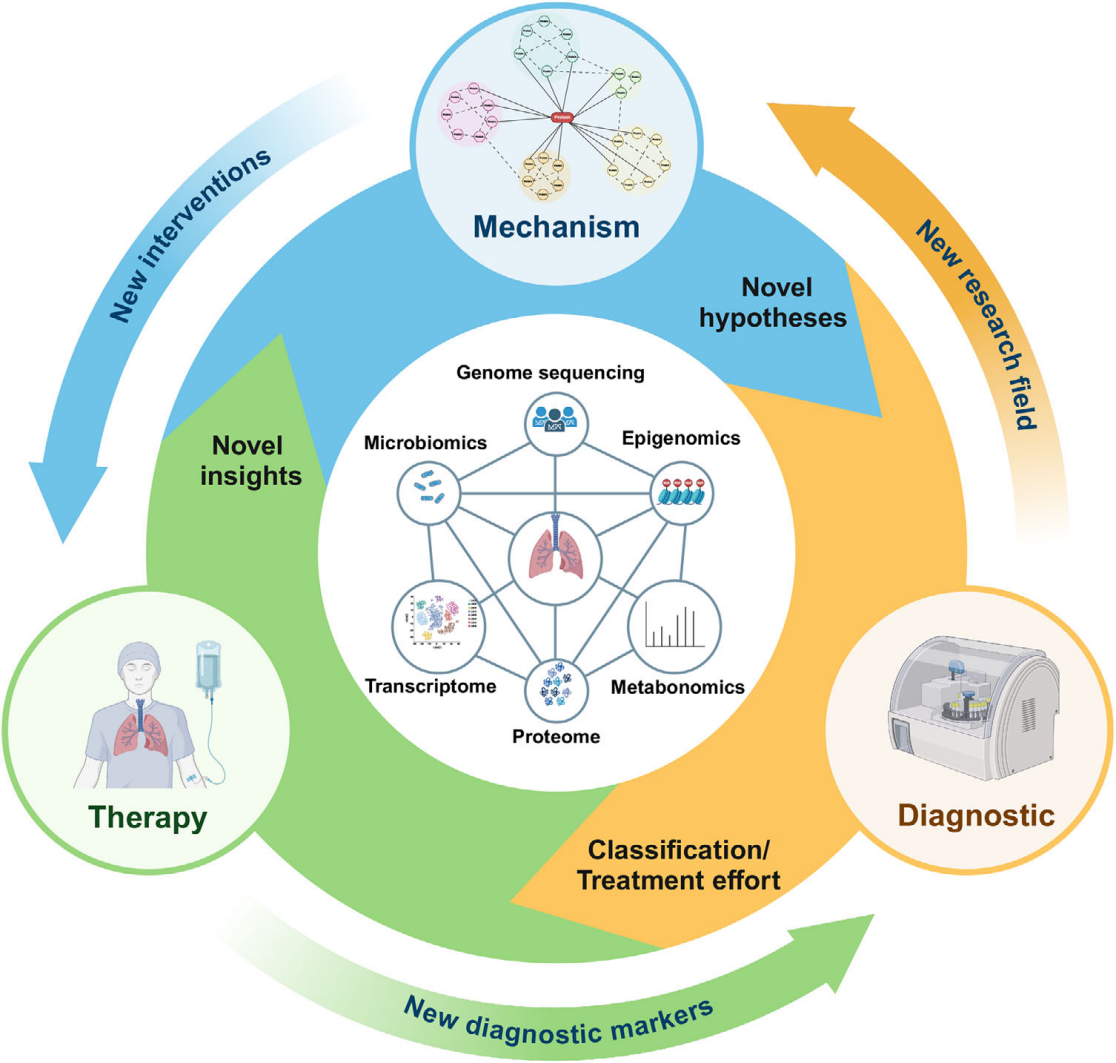
A panoramic understanding of PF toward comprehensive multitargeted interventions. This figure illustrates the interconnected and mutually reinforcing relationship between the study of mechanisms, diagnosis, and treatment of PF. The cycle begins with understanding the mechanisms underlying PF, which leads to the formulation of novel hypotheses. These hypotheses drive the development of new diagnostic tools and classification efforts, enhancing the ability to identify and treat the disease accurately. The insights gained from diagnostics inform therapeutic strategies, leading to novel interventions that improve patient outcomes. As new therapies are implemented, they provide feedback that refines our understanding of the mechanisms and diagnostics, thus continuing the cycle of innovation and improvement. Throughout this process, various omics technologies are utilized to explore and elucidate the complexities of PF, ensuring a comprehensive and detailed approach to its study and management. This figure was designed using BioRender (https://biorender.com/).

This review highlights that PF involves multiple factors, including epithelial cells, mesenchymal cells, immune responses, and microorganisms. These elements interact with and modify various pathways simultaneously, necessitating a systematic and integrative research approach. Future research on the mechanisms, diagnostics, and therapies should incorporate advanced technologies, such as single‐cell sequencing, organoid cultures, and metabolomics (Figure 3). Single‐cell sequencing can be used to identify the unique contributions of specific cell types to the lung microenvironment. Organoid cultures replicate the three‐dimensional structure and function of the lung tissue, providing a more physiologically relevant model for studying disease mechanisms and testing treatments. Metabolomics can reveal changes in metabolic pathways that contribute to disease progression, whereas microbiology can elucidate the role of microorganisms in PF. These studies should be integrated within a systems biology framework to capture the intricate interactions and regulatory networks involved in PF.

Early and accurate diagnosis is crucial for effective management of PF. Future efforts should focus on the discovery and clinical application of new biomarkers to detect this disease in its early stages. Advanced imaging techniques and molecular diagnostics can be used to monitor disease progression and evaluate treatment responses. Reliable biomarkers can facilitate personalized treatment strategies, allowing timely and targeted interventions to slow or halt disease progression.

Because of the multifactorial nature of PF, a single therapeutic approach is often inadequate. Therefore, a combination of treatments that target multiple pathways and cellular interactions should be considered. Combining antifibrotic drugs with cell and gene therapies, as well as leveraging nanoparticles and gene‐editing technologies, can enhance treatment precision and efficacy. Exploring the synergistic effects of various therapies can improve therapeutic outcomes and reduce adverse effects. Supportive measures such as lifestyle modifications, pulmonary rehabilitation, and oxygen therapy should be incorporated to improve the overall quality of life of patients.

In summary, the pathogenic mechanisms underlying PF are complex and involve numerous cellular interactions and pathways. Future research should adopt a systematic and integrative approach to uncover the intricate details of PF pathogenesis. Early diagnosis using novel biomarkers and advanced imaging techniques coupled with multimodal treatment strategies holds promise for significantly improving patient outcomes. By embracing advanced research tools and comprehensive therapeutic approaches, substantial progress can be made in understanding and treating this challenging disease, ultimately bringing hope to patients.

## AUTHOR CONTRIBUTIONS

Yu Zhu, Hongzhi Yu, and Huaiyong Chen conceptualized the manuscript and oversaw the writing process. Jianhai Wang, Kuan Li, and De Hao were responsible for drafting and revising the manuscript. Xue Li contributed by editing the manuscript and compiling relevant research summaries. All authors reviewed and approved the final version of the manuscript.

## CONFLICT OF INTEREST STATEMENT

The authors declare no conflict of financial interest.

## ETHICS STATEMENT

Not applicable.

## Data Availability

Not applicable.
